# Stroke and liminality: narratives of reconfiguring identity after stroke and their implications for person-centred stroke care

**DOI:** 10.3389/fresc.2024.1477414

**Published:** 2024-12-03

**Authors:** Joseph Hall, Frederike van Wijck, Thilo Kroll, Helena Bassil-Morozow

**Affiliations:** ^1^ Department of Media and Journalism, Glasgow School for Business and Society, Glasgow Caledonian University, Glasgow, United Kingdom; ^2^ Research Centre for Health, School for Health and Life Sciences, Glasgow Caledonian University, Glasgow, United Kingdom; ^3^ UCD Centre for Interdisciplinary Research, Education and Innovation in Health Systems (UCD IRIS Centre), UCD School of Nursing, Midwifery and Health Systems, University College Dublin (UCD), Dublin, Ireland

**Keywords:** stroke, liminality, identity, person-centred care, healthcare professionals, rehabilitation

## Abstract

**Background:**

The complex physical, cognitive, and psychological consequences of stroke can disrupt a survivor's sense of pre-stroke normality and identity. This can have a substantial impact on their individual and social lives. Individual reports about life after stroke have improved our understanding of this impact. However, stroke support systems, struggling with increased demands due to a growing stroke population and guideline requirements, require deeper insights based on synthesised narratives into what can enable stroke survivors to rebuild their lives and identities positively to provide person-centred care.

**Methods:**

A qualitative study using Charmaz's Constructivist Grounded Theory (GT) method. Semi-structured interviews lasting 60–90 min were conducted. These interviews were held at least 12 months post-stroke.

**Findings:**

Thirty participants were interviewed from across the UK (14 women, 16 men; aged 31–86; 1–25 years post-stroke). Participants reported the disruption stroke could cause to their sense of identity. The concept of liminality, that describes the ambiguous, transformative state between two distinct stages, where an individual or group exists “betwixt and between” stable conditions, explains the challenge to identity post-stroke. Participants reported developing an uncertain sense of identity as they struggled to structure identity in the same way they did before stroke. This is because the participants' characteristics, traits, hobbies, or future life plans, as well as social relationships and roles, were affected by stroke. Subsequently, participants began a process of reconfiguring their identity, an often-long-term process that involved coming to terms with, and integrating, the impact of stroke on their lives. As a result, participants could enter an indefinite period of sustained liminality as they contend with long-term change and continued uncertainty.

**Conclusion:**

The concept of liminality, which emerged from individual stroke narratives for the first time, conveyed the adaptive and enduring nature of a stroke survivor's journey. Post-stroke liminality may continue indefinitely, sustained by a survivor's subjective individual and social situation. This new insight justifies the urgent call for long-term rehabilitation and support that is tailored towards the unique nature of a survivor's circumstances. Further work is required to understand how tailored, long-term and person-centred support can encourage survivors to positively reconfigure their identity.

## Introduction

Stroke continues to be the second-leading cause of death and the third leading cause of disability worldwide (1). Stroke survival rates have improved due to innovations in the delivery of acute services; however, with ageing populations, and an expanding range of risk factors for stroke, the stroke population is growing (1–3). This is resulting in a global situation in which more people are experiencing the multifaceted, often long-term impact of stroke that may disrupt their individual and social lives.

For survivors, coming to terms with the sudden, often drastic, change they experience following stroke, can result in long-term, existential challenge which may threaten their sense of identity (4). For healthcare professionals to deliver genuine, person-centred care, having insight into the impact of stroke on a person's identity is crucial. Identity can be understood as the traits, characteristics, social relations, roles, and social group memberships that define who one is; identity is also shaped by one's position in time, as one carries a concept of who they were in the past, who they are in the present, and who they wish to be in the future (5) (p. 69). Stroke can disrupt these important strands of identity through: impacted levels of autonomy and independence (6–9); an altered relationship between body and self (7, 10–12); changing familial roles and responsibilities (13–20); losing and shrinking social networks (4, 17, 21, 22); and experiencing an inability or difficulties participating socially, especially through important social roles such as returning to work (4, 9, 23–25). The personal and social worlds, that are essential in helping define an individual's sense of pre-stroke normality and identity, can be shattered, resulting in a significant disruption to the key routes one used to define who they were. This may result in survivors having to face the loss of a former sense of self and come to terms with the need to rebuild a new identity following stroke (10, 26–28). However, rebuilding identity following stroke is often an unstable, long-term process where the self-perception of a challenged sense of identity can persist (4). As survivors come to terms with the significant impact of stroke on their life, it is common for them to experience social isolation (29, 30), as well as long-term psychological challenges, such as anxiety and depression (30–36).

Healthcare plays a vital role in supporting survivors, not only during their acute care, but their transition throughout the various stages of the stroke pathway. The delivery of stroke care strives to adopt a person-centred approach, that seeks to address psychosocial issues and deliver care centred around a person's needs, preferences, and values in the subjective context of their individual lives (37, 38). However, due to pressures on the delivery of stroke care, the holistic reality of the impact of stroke on a person's life is often reduced. Depending on the time post-stroke, the nature of subacute phase of stroke rehabilitation care one receives is often centred around medical and mobility concerns (39, 40). The psychosocial impact of stroke is often not integrated into the care a survivor receives prior to discharge, as important aspects of recovery, such as psychological support, are often lacking (41, 42). When transitioning out of primary healthcare, many often experience a significant decline in support and disjointed continuity of care (43–45). During this period, stroke survivors and their carers can report how a lack of co-ordinated post-stroke care can leave them feeling unsupported (46, 47) resulting in feelings of abandonment (48–51). There have been endeavours to embed a truly person-centred approach within stroke care in order to support stroke survivors in their long-term rehabilitation with the intention of improving adjustment (52–56). However, the COVID-19 pandemic has worsened the lack of provision of, or access to, post-stroke support (50, 57–59). Moreover, the importance of the need to support survivors in their long-term psychological adjustment following stroke continues to be stressed as an essential research priority (60). These circumstances highlight the urgency for new insights into the long-term adjustment to life following stroke and how best survivors can be supported to rebuild their identities positively, in a way that is centred around their needs.

Illness narratives can help to communicate and frame a stroke survivor's holistic circumstances, extending beyond their medical problem, helping to explore existential elements of an individual's long-term illness experience (61). Thus, stroke survivor narratives can provide powerful insight into experiences of recovery that scopes the psychosocial nature of adjusting to life after stroke and the impact to their sense of identity (62–68). In turn, the findings from such narratives can help to display the needs of survivors and where the provision of support and care can be improved to encourage survivors to adapt positively to life after stroke.

The importance of identity following stroke is not a new issue within the field; indeed, various qualitative studies have sought to specifically investigate, at least to some degree, how identity is impacted by stroke (4, 6, 7, 10, 11, 15, 21, 23, 25, 27, 69–73). While conceptualisations of the challenge and adaption of identity post-stroke exist, there are limitations to the scope and depth to which they explain the long-term identity challenge that stroke can pose (74, 75).

Thus, further research is required, not just to understand the immediate impact of stroke on identity but how this adapts and changes over time, as survivors adjust to the long-term effects of stroke. To holistically understand this process and to provide a theoretical map that marries the significance of both the individual and wider social worlds that manifest within a survivor's long-term experience, this study sought to address the following aims:

1. To gather, from a variety of social backgrounds, stroke survivor narratives that shed light upon the dual interaction of individual and social factors that either help, or hinder, stroke survivors to reconfigure their identity positively following stroke.
2. To establish a comprehensive theoretical map, that details the individual and social factors that help, or hinder, stroke survivors to reconfigure their identity positively.

## Method

### Ethics

This research study was granted ethical approval by Glasgow Caledonian University's Glasgow School for Business and Society Research Ethics Committee and the University Ethics Committee on the 25/09/2019 (Ref no: GSBS EC 016). This study complied with the Data Protection Act (2018) and the General Data Protection Regulation (GDPR).

### Study design

This study was a qualitative, constructivist Grounded Theory (GT) (76) study that used semi-structured interviews with 30 UK-based stroke survivors exploring what individual and social factors help or hinder survivors to positively reconfigure their identity post-stroke.

### Participants

Theoretical sampling was utilised as the sampling strategy. This process is a way of collecting data, and deciding what data to collect, based on the categories and theory that are emerging from the data (76–78). The aim of this process is to help ensure that the data which is collected, and the categories and theory which emerge from it, are as fully explored as possible to ensure the theory one generates is fully formed (77). Participants are, therefore, sought who can help further explore certain theoretical strands, coming from the data. Thus, sampling should conclude only when no new emerging theoretical strands, or deviant data sources, can be explored; this point is referred to as theoretical saturation (77).

Participants were approached via community support groups, care homes, and online social media adverts and posts throughout the UK. The inclusion and exclusion criteria were that participants had to be: aged 18 years or above; 1-year post-stroke; living in the UK. Only those unable to provide informed consent, or who had a severe cognitive or communicative impairment restricting participants from portraying their narrative could not be included. Prior to providing written informed consent, participants were given a Participant Information Sheet, clearly describing the aim of the study and topics to be covered. Once consent was provided, participant information was captured in a questionnaire, sent to participants prior to their interview. Further information was gathered in regards to the participants' living arrangements, occupational status, self-perceived stroke severity, self-perceived recovery level, social activity, and level of social deprivation. Stroke survivors with aphasia were supported to join the study through the use of the communicative tool Talking Mat (79); however, no participants required it.

### Setting

Initially, interviews were intended to take place face-to-face. Those who were out of a reasonable travel range were to be communicated with via secure online video platforms or phone call. Secure and encrypted online video call platforms included: Skype^™^ or Zoom^™^. Videocalls only took place privately between the first author and the participant. Consenting participants were welcome to invite trusted family members or friends to the interview, if they wished. Data was only collected from the consenting stroke survivor.

### Data collection

Qualitative research interviews lasting 60–90 min were conducted following the constructivist GT method (76). Interviews were conducted in a semi-structured manner.

GT was selected due to the iterative process inherent within the method, which allows data to emerge organically as data collection and analysis progress alongside one another, mutually influencing the direction of the study (76, 78). This dynamic research process enabled the explorations of the collective relationship between developing themes, providing a holistic representation of life after stroke. Constructivist GT provides an epistemological and ontological foundation which acknowledges the importance of subjective perspectives and social interaction in the construction of social phenomena (76). This allows one to locate and interpret participants' “meanings and actions” which enables one to show “the connections between micro and macro levels of analysis and thus link the subjective and the social” (76) (p. 241). This position enables one to navigate the push and pull between the individual and social, in order to theoretically conceptualise the process of reconstructing identity following stroke.

Initial interview questions were guided by from Ivtzan et al.'s (80) work on second wave Positive Psychology. The core ontological concern of second wave Positive Psychology lies in exploring the dialectical relationship between positive and negative experiences, and how this interaction manifests itself within the individual (80) (p. 6). As a result, second wave Positive Psychology does not focus solely on the psychology of positivity *or* negativity, nor does it view the two as polarising opposites but necessary, often-intertwined, aspects of the holistic human experience. This is of particular importance when observing a personal response to crisis, in which one can transcend the often-anticipated negative outcome, as a person remains resilient to, and even flourishes in spite of, the adversity they face.

The initial interview questions developed from second wave Positive Psychology supported the gathering of a broad range of participant experiences. This framework created an initial platform for the exploration of more in-depth and specific theoretical strands pertaining to life after stroke to emerge from. Moreover, the applicability of this framework was constantly tested against the emerging data. This means that the topics explored, and the questions asked, within interviews had the potential to be altered during the collection process to sufficiently saturate the theoretical strands emerging from the data (76, 78, 81). Therefore, the framework should be considered as a starting point that changed and adapted throughout the iterative nature of the study towards the generation of a novel grounded theory.

The interviews were digitally recorded and transcribed verbatim.

### Data analysis

Data was analysed through the constructivist GT approach. The various stages of coding within this process are shown in Table 1. Throughout the coding process, the analysis was conducted as quickly as possible and generated codes were kept as close to the data as possible (76). To maintain proximity between constructed codes and the data, gerunds were used to construct code (76). Gerunds are noun forms of verbs, such as “revealing”, “defining”, “feeling”, or “wanting”, that help to explain what exactly is happening in a fragment of data (82) (p. 164). Coding in such a way helps to unveil the implicit processes within the data, to establish connections between different codes, and maintain active and emergent analysis (82) (p. 164). Furthermore, the coding process was supported by the application of *in vivo* codes; codes that employ the language used by participants in the raw data, that helped to reflect the original meaning of the data within the code (76). The initial codes collected were then elevated to focused codes by identifying codes that were recurrent or that illuminated significant aspects of the phenomenon being explored (76), and are able to “carry the weight of analysis” (82) (p. 164). These focused codes then formed the preliminary theoretical categories which were then thoroughly explored and tested through the GT method of theoretical sampling and saturation. Case-based and conceptual memos written by the first author also supported the analysis process (76, 82).

**Table 1:** Constructivist GT coding process (76).

Coding step	Description of coding step
(1) Initial coding	Initial segments of data conceptually labelled with codes through line-by-line coding. Important initial categories begin to form.
(2) Focused coding	Initial codes are compared with one another. Focused codes are developed depending on the frequency and significance that initial codes appear within the data. Focused codes become more conceptual, advancing the theoretical direction of the analysis. Higher order categories begin to form.
(3) Theoretical coding and abductive reasoning	Theoretical coding is the process of displaying the relationship between the focused codes that have already been developed. Abductive reasoning supports this process as the researcher makes inferential leaps to explain surprising data. These inferences are then empirically tested against the data until the most plausible explanation is found. The relationship between higher order categories is explained.
(4) Creating grounded theory	Final grounded theory is constructed, evidencing the relationship between the theoretical strands and categories explored over the course of the study.

Credibility and confirmability were ensured through rigorous constructivist GT practices, such as theoretical sampling, verbatim transcription, and *in vivo* coding, preserving participants' original expressions. Dependability was reinforced by the systematic, iterative GT approach, allowing flexibility while consistently tracking themes. Coding stages and memos provided a clear, replicable audit trail, enhancing data reliability.

## Findings

Thirty stroke survivors took part in this study. Pseudonyms are used where participants are referred to directly. No participant that responded was ineligible to take part. The participants composed of: 14 females and 16 males; age ranged from 31 to 86 years, with a mean age of 55 years; time since stroke ranged from 1 year to 25 years, with a mean of 5.4 years; 13 participants lived in Scotland and 17 in England, no participants were from Wales or Northern Ireland. In terms of living arrangements, just over two-thirds lived at home with family, five lived at home alone, whilst two lived in care homes. In terms of occupational status, just over a third were retired, while a third were either employed part- or full-time, three participants were on long-term sick leave, five were unemployed and one participant was studying. The sample included a wide range of self-reported stroke severity levels from mild to severe, with the majority reporting their stroke as being “moderate”. Regarding self-perceived recovery level, the majority described their recovery as “good”, with only four participants describing their recovery as either “poor” or “no change”, and one did not respond. None of the participants responded saying that their level of social activity had increased following stroke, with 18 saying their social activity had decreased and 12 saying they were as socially active as compared to prior to stroke. Finally, in terms of the level of social deprivation (1 being high and 10 being low), the levels captured ranged from 1 to 10, with the mean level of social deprivation being 5.

Ten face-to-face interviews were conducted before the COVID-19 pandemic required the study be carried out remotely. The final 20 interviews were conducted through an online video call platform or through a phone call, as per participant's preference.

### Post-stroke liminality and identity reconfiguration: a conceptual model

**Figure 1:**
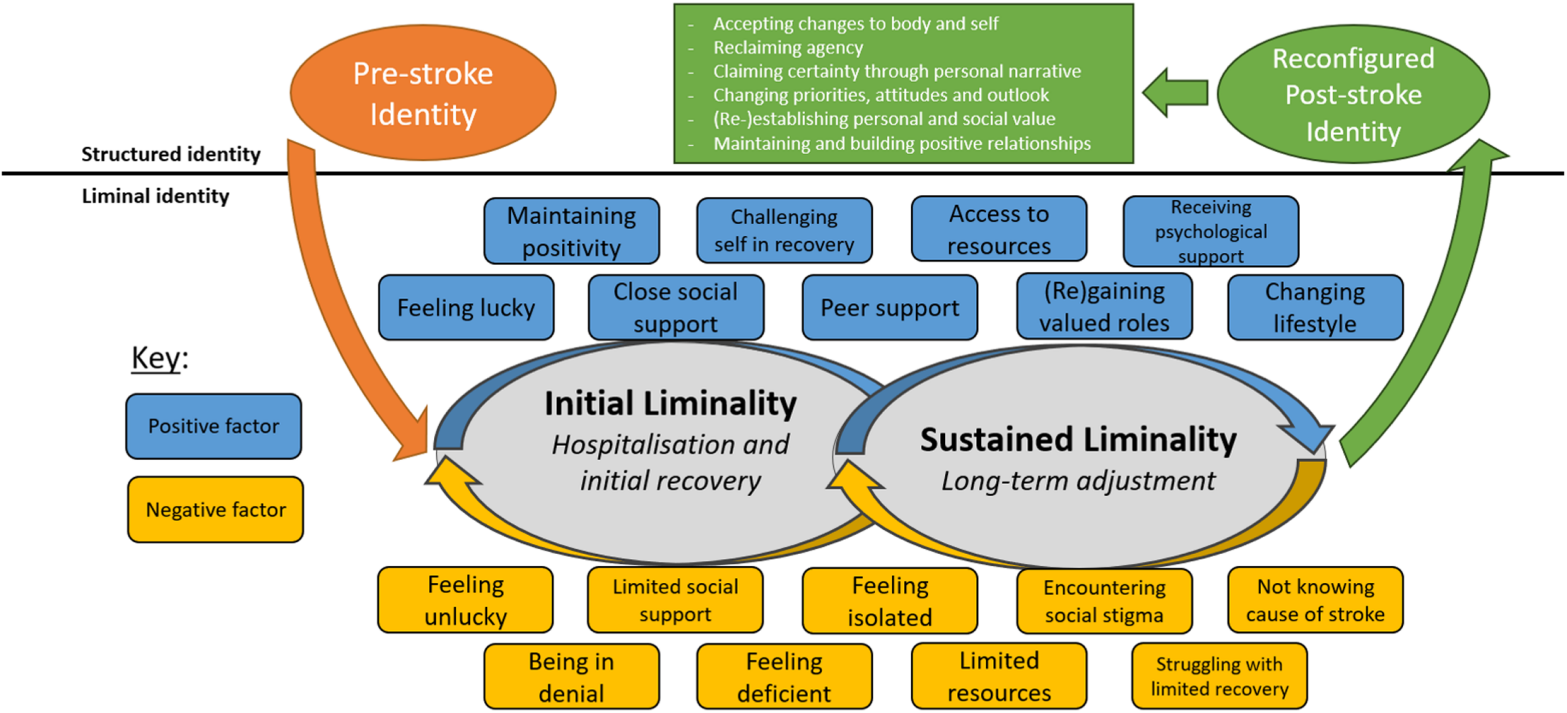
Conceptual model of post-stroke liminality and identity reconfiguration.

The conceptual model, generated through this grounded theory study, is shown in Figure 1. Liminality is at the core of the model, providing a framework to understand the uncertain, transitional nature of identity following stroke. Liminality was first detailed by van Gennep (83) in the early twentieth century, and was adapted and returned to prominence by Turner (84). In its infancy, the concept of liminality focused predominantly on defining the ambiguous and non-structured middle phase of a rite of passage (or ritual process), during which participants lose their pre-ritual status but also do not yet hold their post-ritual status (83). During the liminal phase, participants “stand at a threshold” (83) (p. 21) between their pre-ritual and post-ritual forms of constructing identity, time, or community. The concept of liminality has been applied to understand a variety of chronic illnesses, including: cancer (85–97); kidney disease (98); chronic pain (99, 100); dementia (101); and chronic fatigue syndrome (102).

Participants reported a near-total fluctuation in the way they lived their lives, as they experience instability and change in how they perceive themselves, relate to the world, recover and rehabilitate, and engage socially post-stroke. The disruption caused by the universal uncertainty that followed stroke could result in survivors experiencing a state of enduring flux, as aspects of their daily living and identity remain unachievable, ambiguous, or unstable. It is here that the concept of liminality can be applied to explain the way in which survivors feel trapped betwixt and between the worlds of the pre- and post-stroke, as one can feel neither one nor the other (84).

As has been represented in the model, the liminal world one can enter following stroke was often defined by two periods of liminality; an initial period and a sustained period. This concept adapts Little et al.'s (92) work analysing cancer narratives, in which they identified an acute phase of liminality defined by the immediate impact of diagnosis, and an enduring, potentially indefinite, phase defined by a fluctuation between illness and health; recurrence and remission. This concept can be utilised to explain the way in which identities transitioned for stroke survivors throughout their post-stroke journey. The first phase of liminality has been defined as “initial” rather than “acute” as, in the case of the participants, this initial period of liminality can extend beyond hospitalisation, into their initial recovery period and experiences outside of primary healthcare. The length of each period of liminality, and the point at which one transitions from initial to sustained liminality, are inherently indeterminate, often depending on myriad factors, subjective to a person's individual, social and environmental context, which are evident in Figure 1. However, generically, this initial phase seems to help define one's experience during hospitalisation and their initial recovery and return home. The sustained phase of liminality then seems to extend beyond this period, into the long-term experiences of survivors, potentially continuing indefinitely, as survivors grapple with the continuing effects of stroke on their lives. During sustained liminality, participants reported finding themselves trapped between their previous pre- and post-stroke identity, as many struggled to re-engage successfully with society and aspects of their pre-stroke lives.

As can be seen, liminality was often reported to end once a survivor had reconfigured their identity after stroke. Findings suggested that, at the core of this transition, is a long-term existential journey in which survivors psychologically re-evaluate their priorities, life goals, beliefs, social relationships, and life narratives. Each are core pillars of an individual's sense of identity; as these components shift, so does one's identity.

When viewing this novel theoretical model, the challenge to, and the process of reconfiguring identity must be acknowledged as inherently nonlinear. Many survivors may navigate these various periods of liminality, but the time and stability in which they remain is often difficult to define and variable. The presence of instability, uncertainty, and thus of liminality, appeared to be persistent throughout a stroke survivor's journey, as survivors reported fluctuating between improvement and vicissitudes throughout their long-term adjustment.

### Initial post-stroke liminality

As evidenced in this study, stroke survivors were often unprepared for the immediate challenges they may face following stroke, as their personal and social worlds can be significantly disrupted and the ways one establishes their identity are inaccessible, often resulting in the formation of an uncertain, liminal identity post-stroke.

#### Experiencing initial challenge to identity

For the participants of this study, the separation that took place from pre-stroke identity was abrupt, unexpected, and out of one's control. This period of separation is an often-forceful uncoupling from a person's sense of normality and self, as individuals must often fight for survival and can acquire a variety of multifaceted impairments and activity limitations. Helen helps reflect this initial period of uncertainty, as she says:

“It’s like having your life pulled out from under you. […] Your brain is your sense of identity: what you can do, how you can read, you know, how you can think. I was proud of that. […] [what I was worrying about most was] that I wasn’t me anymore, that I didn’t have a, kind of, future. I did..I worried most about getting back to work. That was a big thing.”—Helen, aged 60

Following stroke, Helen expressed the fundamental challenge stroke posed to her sense of self, as she felt that her brain and, as a result, her sense of identity, were under attack. This created concerns about what the lasting consequences would be and what impact stroke may have on her future. This was a common concern for study participants in the immediate aftermath of their stroke. Indeed, the initial disruption caused by stroke has very real implications for stroke survivors, as their body can no longer reflect their established sense of identity. We see this reflected in Ewen's experience, as he says:

“It was a bit strange, I mean I, I kind of, very quickly regained my ability to walk but like the—I still had the weakness in my left side and down my left arm, so, trying to do the sort of stuff we’d have done about the house before was really fatiguing and quite difficult to do that. I said the, kind of, the sudden reliance on my wife and children to do stuff that I would normally have done myself. […] I've always been really independent so that—having to ask people for, for lifts you know, to come and collect me, or to take me to places, is something that I’m, I still strugglin' with.”—Ewen, aged 47

Ewen's experience reflects the often-drastic reduction in one's autonomy and independence following stroke. This change can challenge a person's established identity and normality, through limiting their capacity to work or role within the family.

#### “The feeling of powerlessness”: experiences of interacting with healthcare

As survivors experience a shaken sense of self, often carrying concern about what life will be like following stroke, their experiences within the institution of healthcare can set expectations and limitations of what it means to live with stroke. As a result, the very way in which individuals navigate the cultural and environmental experience of healthcare may support the expectations survivors develop for life after stroke.

Noteworthy was the finding that, in 13 out of 30 cases, stroke was not immediately considered when participants initially interacted with HCPs and their condition was misdiagnosed. In the participants' experiences, misdiagnosis seems to be led predominantly by HCPs' assumptions about the expected presentation of stroke. However, as was the case in this study, many stroke survivors experience varied symptoms of stroke that do not always fall within this bracket. As can be seen in Jessica's experience:

“The paramedics came, and they did like a FAST test, you know, when they're asking ya to lift one arm up and all this stuff. […]. And I knew that I was FAST negative. […] And they actually said go back to bed and get your doctor when he finishes his work. […] But I was saying, “No, I need to go to the hospital.” […] Even the doctors will still say, “You’re young to have a stroke.” It's unbelievable, I don’t know what workbooks they’re working from, but they all seem to think you need to be old to have a stroke […] So I wish [HCPs would] stop associating [stroke] with old people because, for people like me, that can stop you getting the help you need straight away. Because of me age, they didn’t think I was having a stroke, they thought I was having an ear infection. Maybe if they hadn’t had put this age stigma on us straight away, they might have treated us how they needed to.”—Jessica, aged 33

Jessica believes that age-related assumptions and not showing common symptoms resulted in delays before accurate diagnosis and receiving critical care. It was common for participants to cite that they believed their input and voice was not given the same weight as that of the HCPs they interacted with. Participants reported experiencing a power imbalance between themselves, as patients, and those who cared for them. Helen helps to explain how this weighs on a patient:

“The feeling of powerlessness, and being at the mercy of all these medics, was phenomenal. […] I didn’t really think I was involved in any real decision making […] I kept asking for some kind of..I knew that I needed some kind of psychological help, you know, and there..there was no one available […] [Healthcare staff] were always telling me things, and that's the only thing I would say about care, no one actually asked me what I felt. And so, you know, they just tell me, “Oh, you're very low. You mustn't be low.” And I'm, like, it was really unhelpful. And these people are wonderful people…they were very kind and they tried.”—Helen, aged 60

Study participants often felt as if their agency over their own health was being diminished. However, it is important to state that survivors did speak positively about their interactions with HCPs. These positive experiences were often defined by HCPs acknowledging the power dynamic that is at work within the healthcare setting and aiming to redress the balance by providing patients with greater agency. This can be seen in William's narrative:

“[Healthcare staff] had time to talk to you and nothing was too much bother […] you could not fault [the HCPs] at all, they gave me, they gave me an illusion of independence and freedom, they were friendly, they were knowledgeable, they were helpful.”—William, aged 63

HCPs played a valuable role in supporting William's agency and providing him with a sense of control and involvement throughout his hospitalisation. In fact, even within negative experiences, such as Helen's, she states that the HCPs she interacted with were well-intentioned and acknowledges their attempts to be supportive. What is clear is that the difficulties many encounter are often not due to bad-faith actors but the way in which institutional and cultural practices create an unhelpful dynamic between patients and practitioners. This produces a discrepancy between service provider and user, as the concerns of the participants were often of a psychological nature, whilst HCPs were reportedly preoccupied with diagnosis and survival strategies initially, and with physical/cognitive rehabilitation later on.

As Elodie helps to explain, participants’ experiences of healthcare often fuelled a loss of faith or changed perception of the epistemologically heightened position of healthcare:

“Ive learned that it's not because someone is a doctor and they act with confidence that they actually know what's going on. Because the doctor who told me I had special cancer, breast cancer, was very, very confident when he told me that. […] And he talked like—we were in a hospital, he was wearing, you know, all the adequate outfit for a doctor. I had no reason not to trust him, you know? He's in a position of power […] They are not God is what I've learned. They are human, like you and me, and they are doing their best and..they do [make] mistakes, as well. And I say I didn't..I didn't lose respect; I lost a bit of trust. I've learned I need to be my own advocate. If I want something, when it comes to my health, I am the one in charge. I don't want to be a passenger in my own body.”—Elodie, aged 32

As can be seen in Elodie's quote, many participants reflect on the importance of individuals taking charge of their health and decisions made regarding future treatment. Despite the wealth of medical knowledge HCPs may have, participants reflect that they now see themselves as the ultimate authority on their experience and health. As a result, this change in perception is a reclamation of agency in the sphere of healthcare, as participants challenged the heightened epistemological position of healthcare and HCPs, defying the deference patients are often-expected to display within the healthcare system.

Study participants reported finding healthcare environments difficult following stroke. This was often an issue for younger survivors, as they experienced care that is orientated towards, predominately occupied by, and named after, the elderly. This can have a negative impact on the way a survivor sees themselves, as Helen states:

“When I got [to the rehabilitation ward], I was with stroke people and they were all old. You know, a lot of them were incontinent, they couldn't talk, I mean I just—that was awful…I felt ancient *(laughs)*, and so I wouldn't let people see me there for a while; I mean, my closest friends came, but I didn't want anyone from work to see me amongst all these old people. I was in a bed next to this woman who had dementia…I found that really depressing […] I felt like I am old, especially after being…with all these old people because it was horrible, that kind of stuck with me. So that compromised…my sense of my attractiveness, definitely.”—Helen, aged 60

Helen wanted to distance herself from being associated with these possible outcomes of stroke and thus restricted who she would allow to visit her while she was in hospital. Yet, the experience of being in this environment has been internalised by Helen, having a long-term, negative impact on how attractive she believes she is. Helen's experience highlights how one's hospital experience can become representative of the sudden discrepancy between a person's pre-stroke sense of self and a sense of self that is destabilised following stroke. At this crucial time, the labels that are attached to an individual, and the visual information they absorb while in hospital, may help support survivors to construct negative reflections on their identity following stroke. In all, survivors can experience a limited sense of agency and voice, something that is not only reflected in their newly acquired impairments and vulnerable health, but also within the status they are provided by the system, the environments they experience, and power structures they must negotiate that are embedded within healthcare.

#### Self-body disruption

Stroke often resulted in global disruption to participants in their daily lives, in large part due to their reduced ability to physically engage with the world around them. This disruption was severe enough that they began to feel a disconnect between their self and bodily experience. We can see this in Peter's case, as prior to his stroke he “was married with a young child” and describes himself as being in his “early-mid thirties”, being “fit and healthy” and that he “played sport to a decent level”. However, following his return home following stroke, he found adjusting difficult:

“There was an awful lot of frustration from my point of view of not being able to do the things I did before, or getting very, very tired from doing the things that I did before.”—Peter, aged 57

Peter's experience reflects Ellis-Hill et al.'s (7) exploration of the self-body split in survivors of stroke, in which they found that survivors would experience a long-term struggle with a body they found to be separate, precarious, and unreliable. In Peter's case, which was shared across many of the survivors who took part in this study, the unreliability of his body and his incapability to engage in the things he used to do prior to stroke was a source of major frustration for him. This fragmented and disconnected existence from his pre-stroke self was not short-term, as Peter describes an “18 months to 2 years” experience where he felt an absence of personhood:

“I see pictures of myself and it's my body there, and there is a body there, but it looks as though—and I feel a bit silly saying this—but it looks as though there is no soul in that body. It is just, there's a shell, it's like looking into hollow, hollow ice. There's nothing there, there's no sense, there's no feeling. And that's how I felt as a person, I felt, I’m here and here's a body but I'm not here, I'm not part of it.”—Peter, aged 57

In Peter's case, we do not only see an individual struggling with a body that sits in opposition to one's established sense of self, but a reflection that the devastating change he experienced destabilised his identity to the point at which, upon reflection, he did not feel present in his own body. This conflict between a drastically altered body and a person's sense of identity that sits in opposition, is often what drove the existential identity crisis following stroke that survivors experienced, as one feels caught betwixt and between contrasting realities.

#### “Why me?”: experiencing and resisting self-pity

As time progresses, stroke survivors are often left with the prospect of long-term challenges that continue to hamper their ability to participate in crucial activities that help form their identity (6, 7, 21, 75, 103). This period of realisation was often one of the most challenging psychologically for the participants, and marked a significant moment for survivors in their attempts to come to terms with the impact of stroke. Diane helps elucidate how a survivor may feel at this time:

“It dawned on me that this was no quick fix and that it would probably take quite some time and that, you know, it's not a good thing to contemplate really, is it? You know, so I, I think I had a sort of week or two where I was really just upset and then I suppose, you know, like all the things that people go through, you know, like [thinking] “Why is this happened to me? Why should I have had a stroke?” but, you know, why not me? So yeah, I think the first sort of couple of weeks were probably quite difficult, psychologically more than the physical side of it.”—Diane, aged 62

The psychological response of self-pity, feeling unlucky, and questioning “why” stroke has happened was common among the participants. Seemingly, this response is a reflection of the possible devastation caused by a survivor's autonomy and capacity reducing. It is during this period that survivors were faced with either the immediate aftermath of stroke when survivors' conditions and capacities were at their worst, or when recovery stagnated and the long-term impact of stroke began to set in. In participant narratives, this was often the starting point of a long-term process of ruminating over, and potentially coming to terms with, the impact of stroke and a forcibly deconstructed sense of identity. While participants, such as Diane, state that their most challenging psychological period was during the early stages of their post-stroke journey, the psychological impact of stroke will likely persist for most, as Lawrence attests:

“Psychologically, for me, for a long, long time the major issue, the stumbling block for me, was trying to think to myself why the stroke had happened to me, like the ‘why me?’ issue.”—Lawrence, aged 58

Lawrence's experience is one that resonated with many of the participants of this study, as the enduring psychological impact of stroke was frequently stated as the most challenging aspect to adjusting to life following stroke.

### Long-term identity discrepancy and sustained liminality

As is reflected within the conceptual model (see Figure 1), determining when initial liminality ends and sustained liminality begins is inherently difficult. This is because the very nature of sustained liminality is the continuation and evolution of the challenges one faced within the initial stages of liminality. It is during periods of sustained liminality that one begins to grasp the full extent to which stroke has impacted their capacity to interact with roles and responsibilities that play, or played, a key role in establishing a sense of identity. Within this phase of liminality, participants reported attempting to re-engage in meaningful ways with society. As survivors attempt to expand the boundaries of their capacity and responsibilities following their initial convalescence period, many of the barriers that restrict their ability to do so became apparent. Resultantly, the discrepancy between an individual's post-stroke reality and their identity prior to stroke often defined the psychological difficulties that were posed to them. It is this existential and fundamental change for survivors that can result in a long-term, and potentially indefinite, period where survivors attempt to come to terms with losing a valued sense of identity.

#### Continued long-term identity discrepancy

For many participants, uncertainty drove the indeterminate nature of life after stroke, often meaning that there was no eureka moment in which one realises that pre-stroke normality is no longer feasible. Participants reported this change as a result of a slow psychological process that has many twists and turns, based heavily within the subjective context of each individual. Elodie helps to explain this:

“The world has moved on and I keep thinking, “Why haven’t I?” […] When does this post-stroke you become the new you? When do you stop trying to recover? When do you stop trying to get back to what used to be, the normal you, and you just accept that this is the new normal you?”—Elodie, aged 32

What can be seen here is Elodie portraying the sense of inertia she felt following her stroke; as life moved on, Elodie felt stuck while she processed her experience. Elodie can be seen questioning the exact timescale of recovery and identity reconstruction following stroke and her shift in focus from returning to her pre-stroke identity vs. acceptance of a new identity. What Elodie helps to portray is the complex process that emanates from the way stroke challenges identity and the inherent dissonance between new and old aspects of identity. The inertia Elodie speaks of can be seen across many participant experiences, this reflects an inability to return to pre-stroke life, which leaves survivors questioning their sense of identity. Furthermore, the line between “old” and “new” identity is an often-unclear one, in which some elements of identity are lost and some remain, meaning that the construction of a completely new identity post-stroke is not what occurs but a recalibration between old and new. It is through this ambiguity and uncertainty in self that the liminal and unstructured experience of the participants was sustained.

#### Social re-integration and stigma

Returning to work was a core goal, at least initially, for many of the participants of this study; however, most were unable to successfully do so. This can be seen in the case of Peter, as he lost his job following stroke and had continued difficulties maintaining permanent employment. He explains how he struggled against the expectations he had of himself, and the expectations he believed others held for him:

“Well, I lost my career, I lost my purpose at the time […] I became very, very frustrated with things very easily, at the time I had very little patience on myself and others. My expectations of myself and others. I’ve always had high expectations of myself, I felt, felt that I couldn’t achieve what I expected of myself. Which, again, is probably part why I come back to what I mentioned earlier about feeling like a failure because I wasn’t achieving what I expected of myself and what I thought others expected of myself.”—Peter, aged 57

Peter's experience helps to show the deficiency one can feel following stroke as they fail to successfully meet the pre-stroke standards they have set for themselves in regards to their general capacity, roles, and responsibilities. Peter describes losing his sense of purpose and feeling “like a failure” due to his inability to meet pre-stroke expectations. Peter often encountered a lack of understanding from his close social support network, ultimately resulting in the collapse of his marriage. Furthermore, Peter goes on to describe feeling that he only recently rediscovered a sense of purpose, twenty years following his stroke, as he has engaged in charitable endeavours and “doing something for the wider good.” This example shows the indeterminate and potentially indefinite experience of identity discrepancy following stroke the participants reported. Here one can see how a liminal identity can be sustained as stroke survivors may struggle to re-engage with society, which is reflected within the conceptual model (see Figure 1).

Stigma played a notable role in the experiences of the participants of this study. The participants detailed varied instances of how stroke-related stigma often occurs not just on an interpersonal and structural level but on an individual level too. Stroke, and its various invisible and visible consequences, often mean that individuals encounter stigma that extends beyond the boundaries of stroke-specific stigma, as judgements encountered are often surrounded by wider societal stigmas that relate to issues such as: mental illness, disability, and brain injuries. Ultimately, stigma plays a significant role in erecting barriers between stroke survivors and society, sometimes preventing survivors from successfully reintegrating into society.

For participants who had physical disabilities, wider social stigma pertaining to disability could be seen to impact their experience. Jessica describes in her narrative how the social labels attached to disability can persist, reducing the identity of an individual to negative assumptions about disability. This can be seen as she states:

“I decided I wanted to, you know, be that person that people were inspired by and didn't feel sorry for. […] I think everyone's always got that at the back of their mind, that I am disabled. So, no matter how well I get, I think everyone will always think of us as being disabled. I think people pity you more as well. And I don't know why, because I lead quite a positive attitude and I think I don’t give people reason to pity us but I think people do pity you. I mean, I was at a wedding […] And I felt nice about meself, I was all dressed up and that. And I remember someone sitting down and saying, “Just looking at you, you wouldn't think there was anything wrong with you.” And she meant no harm by it, but it really upset us [..] People think of you as having something wrong with you and I don't think I've got something wrong with us. I'm just—I can't use me arm, but I'm still me…I do think people think of you as not being a full person.”—Jessica, aged 33.

Here we can see Jessica's belief that she is predominantly labelled by others as being disabled, and that, even as time moves on and she adjusts positively, she is unable to overcome this label. Jessica describes the discredited status that comes with such a label. Indeed, while Jessica rejects the pity that was placed upon her, as she strives to be a person that is driven to inspire others and live positively, she acknowledges that she may never be able to shed the labels attached to her by others.

For those with invisible impairments following stroke, they could often encounter a lack of understanding from others as to the nuances of their impairments. This was a common frustration for many of the participants, as the inability to understand, or even perhaps acknowledge and take seriously, the internal wellbeing and capacity of an individual could be a constant struggle for some individuals. Amy helps reflect these issues, as she explains:

“The things that have brought me down is people just not understanding brain injury and that's my friends, my family, my work colleagues. You almost have to explain it to them [the impact of fatigue] and even then, they’re like, “Yeah but I feel tired sometimes” […] Fatigue and anxiety are ultimately two invisible things, they’re not a crutch, they’re not a wheelchair, they are, they’re just not evident and that's quite difficult […] This is where I think in part it's about withdrawing, you know, maybe that was my way of protecting myself; if I felt like somebody would lack understanding, then I withdrew.”—Amy, aged 45

As Amy discusses, the lack of understanding one can face in regards to the invisible consequences of stroke can be widespread throughout their social network, from family to the work place. This creates difficulties for individuals interacting with society as, if others are not able to understand the nature of one's condition and its consequences, they cannot understand a person's capacity or limitations.

Indeed, stigma and difficulties due to their status as a survivor of stroke were often anticipated by the participants, creating a dilemma for those with invisible impairments whether to disclose their stroke or not. Diane chose not to disclose her stroke, as she believes she would have been seen as a deficient prospective candidate:

“I do really feel that employers, they can pick and choose can’t they? And if you had somebody who was perfectly healthy and somebody who’d had a stroke, I would, I'm sure I would do it myself, I'd take the perfectly healthy person. So that was the reason I didn't actually want to disclose to them.”—Diane, aged 62

Diane's quote cements the theme of deficiency that runs through much of the judgement, or anticipated judgement, stroke survivors spoke about within their stories. It is for this reason why some participants choose not to disclose their stroke, as it is an attempt to resist and avoid stigma associated with stroke.

Finally, self-stigmatisation can be engendered in an individual as they attempt to reintegrate into the wider world. Emma found the transition from hospital to returning home difficult, as she was unprepared for the change in social interaction she experienced:

“When you’re in hospital, you’re in that setting where people expect to see people in wheelchairs or poorly people…when you go into the outside world…people are looking at you differently and just, it was a massive culture shock, massive…as kind as people are, you feel that, I don’t know, you’re being [treated] differently and for me it was—I just hated it […] I should be able to walk…do things myself…I felt ashamed for people to see me, like from how they’d seen me maybe a year ago, running about, you know, to…in a wheelchair.”—Emma, aged 33

The institutionalisation Emma experienced left her feeling unprepared for the outside world and the shift in social interaction she would face. Emma believed that the use of a wheelchair represented a lower form of existence, beneath her pre-stroke autonomy and capacity, resulting in her withdrawing from certain social situations; her wheelchair became a visible label, emblematic of her assumed change in status and capacity. Emma's quote reflects how many participants experienced self-stigmatisation and the expectation of how they expected others would react post-stroke.

### Reconfiguring identity following stroke

While stroke survivors often must contend with a range of multifaceted impairments that vary between each survivor, at the core of most of the participants' experiences was the life-threatening nature of stroke and the need to come to terms with one's mortality. It is within the wake of the often-severe, life-threatening nature of stroke that survivors experience a complex existential challenge, one that triggers a long-term existential process that can engender a reconsideration of one's priorities, personal philosophy, and what they believe are the core components to their identity. These psychological shifts are seemingly essential to one reconfiguring their identity and exiting a liminal sense of self. This section explains the resolution of this process and what ingredients seem to indicate a survivor has positively reconfigured their identity following stroke, as seen in Figure 1.

#### Post-stroke acceptance and changing priorities, beliefs, and recovery goals

An important step for many survivors to shift their perspective was to come to terms with the reality that they would likely be unable to return to pre-stroke normality. This is a process that can continue indefinitely, as survivors navigate life post-stroke and a greatly impacted sense of identity. Survivors can be caught in a state of ambiguity as they aim to return to, or mourn the loss of, their pre-stroke identity. It is within this period that feelings of comparative deficiency and self-pity are prevalent. In order to overcome a negative mindset, study participants often needed to separate from unattainable aspects of their pre-stroke identity. Ewen summarises this change:

“I’ve got an opportunity to just refocus my life and go and do something different, you know. And just see this as an opportunity to—that chapter in my life's closed, let's move on and write a new chapter in my life. […] I would say it's only been in the last maybe three or four months that I’ve mentally kind of adapted to it, you know, that's kind of moved away from recovery goals that were very much focused on getting back to what I was and actually come to terms with, I’m a different person now […] important recovery goals are the ones that are about, you know, getting a quality of life rather than, you know, doing the things I used to do.”—Ewen, aged 47

Ewen acknowledges that his previous life before stroke is no longer attainable and shows acceptance of this. He speaks proactively about the opportunity this presents for him to start anew. Ewen's focus shifts to post-stroke life and his goals shift to align with his current capacity following stroke. As Scobbie et al. (104) explain, the challenge of recovery goal adjustment or disengagement following stroke varies depending on whether one can shift their focus to other important goals which are more attainable, and the degree to which potentially unattainable goals are important to re-establishing one's sense of self. What can be seen through the experiences of the participants of this study is that the barriers they encounter can engender a wholesale shift in perspective, as survivors reconfigure their perception of the post-stroke recovery journey.

#### Rebuilding meaning and narrativizing stroke

The experiences of the study participants show that stroke often severs the connections in one's life story but, as one shifts their beliefs, they are able to make sense of and integrate stroke within their life narrative. As a result, stroke is no longer a random, chaotic severance from normality and identity, but a catalyst of necessary change and personal evolution. Stroke survivors may then share this narrative through telling their own stories, and examples within this study could be seen when survivors encapsulated their experience into metaphorical vignettes. An example of this can be seen through Lawrence:

“I described myself feeling like I was in a crater after a bomb had gone off and there's people looking into the crater just after my stroke. And course, as I was getting better, I’d start to come out of the crater and there'd be debris everywhere; debris from the family's thoughts, debris from my relationship, my job. And then it would get to the point where later on the crater starts to grow over and this is the bit where people don't realise there's a bomb gone off, i.e., didn't realise you'd had a stroke. But I know that even though there's green grass over the top of the crater, that bombs always gone off. And it's quite a hard thing to describe cold but that, even the bit about the crater feels different now. Like the bomb crater that I had when I had my stroke, now I'm like, sort of, half a mile down the road from it rather than just having emerged out of it.”—Lawrence, aged 58

Narratives such as Lawrence's reflect how survivors integrate, capture, and make meaning of their experience of stroke within their life narrative. As Pietla et al. (94) discussed in relation to male experiences of prostate cancer, the participants similarly create certainty through narrative closure, helping to compress the myriad uncertainties that are triggered by the impact of stroke. This is critical as the participants' experiences of life after stroke were often defined by the way in which they managed, integrated, accepted, and potentially overcame the uncertainty they face that was produced by the fundamental challenge to identity. To do so, one must often recalibrate central pillars of their identity to align with a newly limited body and a reconsideration of life priorities, goals and beliefs that provide one with a sense of meaning and value following stroke. Ellis-Hill et al. reflect the importance of continuity in a person's life narrative, as they state: “coherence and stability is created through stories. Continuity is essential for psychological well-being and personal integration and for an individual to experience him or herself as one person, despite change and disruption, throughout the life cycle” (74) (p. 153). Thusly, the recalibration of one's life story can be seen as a significant step in enabling the re-establishment of coherence and stability in one's sense of identity, allowing one to reharmonise their past, present and future. As can be seen in the conceptual model (see Figure 1), restructuring one's life story brings liminality to an end, as stroke is integrated into an individual's identity and their focus is centred on life after stroke.

In summary, stroke is a catalytic force of change; one that can fundamentally challenge the presentation and conception of self, the shockwaves of which significantly impact identity construction internally and externally, individually and socially. Regardless of the severity of one's condition following stroke, there is a seemingly ubiquitous process in which one must come to terms with what it means to have had a stroke. At the core of this is an existential journey that often results in individuals facing their own mortality; questioning “why” stroke has happened to them; and re-evaluating their lives in terms of their priorities, beliefs, and life goals. Subsequently, this results in some participants reporting a global shift in core pillars of their identity. It is for this reason that, for many, the existential journey they experience following stroke results in one not solely coming to terms with the impact of stroke itself but rather coming to terms with a new understanding of what it means to be alive.

#### The importance of professional psychological support

The ability to reflect positively on life post-stroke and to reconfigure one's identity are not outcomes that were inevitable for most survivors. A factor of often-singular importance for some participants that encouraged a change in their outlook following stroke was receiving psychological support from professionals. Here, Emma highlights this:

“I really went through a bad stage where, worrying about the future was a huge thing, not necessarily worrying that I would have a stroke again, but just you know, “What's going to happen? How am I going to manage?” you know, obviously I’m gonna, “[Is] the rest of my life is gonna be like this? How am I gonna kind of get by, you know, how am I gonna cope? How am I gonna manage? How am I gonna provide for myself?” That was a huge thing, and I seen, I was referred to a clinical psychologist in the stroke team, and we did a bit of Acceptance and Commitment Therapy, and that was quite helpful to, kind of, bring you back to like the here and now, and not to think, dwell about things in the future, you can't change it. So, I don’t think now, that I necessarily worry about anything like that, I just try to take one day at a time.”—Emma, aged 33

As can be seen, the support Emma received helped her to manage the overwhelming concerns about her future, encouraging a change in her temporal focus to what she can control within the present. While the context varied in which psychological support was provided to participants, for those that received professional psychological support, it often played a significant role in encouraging a change of perspective. Potentially, as shown in the conceptual model (see Figure 1), such support can engender individuals to leave a state of liminal identity following stroke, helping to support them positively reconfiguring their identity post-stroke. The role of peer and social support should not be overlooked here as well, as they were often important in supporting a positive adjustment.

## Discussion

The novel conceptual model detailing the identity reconfiguration process post-stroke, and the application of liminality to understand this process, provides new insight into the ambiguous status stroke survivors occupy as a result of disrupted identities. While the disruption to identity and life narratives has been conceptualised in previous research (74, 75), liminality helps to further explain the transitional period stroke survivors are often thrust into following stroke, as they must manage a disrupted sense of pre-stroke identity which is likely difficult to recreate. Survivors often find themselves suddenly caught between two states of being, the pre- and the post-stroke. Central to this understanding of post-stroke liminality is that the resolution of it and the reconfiguration of identity is not inevitable or guaranteed. Survivors can find themselves in an enduring state of uncertainty that can persist indefinitely.

In all, survivors' attempts to rationalise and make sense of their experience of stroke was often at the centre of their psychological journey following stroke and the process of reconfiguring their identity. While the process of identity disruption following stroke is a well-known phenomenon, it is important to understand the context that surrounds the subjective experience of survivors. As a result, the model developed enables a holistic understanding of the process of long-term identity reconfiguration after stroke, through highlighting the dual importance of both the individual and social worlds of stroke survivors. As such, stroke survivors' experiences are situated within context that is multifaceted, and it is necessary to understand the influence of an individual actor, their social environment, and wider societal structures on any given experience of life after stroke. Through the participants' narratives key factors which encourage survivors to positively reconfigure their identity, or conversely that help sustain post-stroke liminality, have been identified. As a result, this model offers a way to conceptualise the transitional process of the pre- to post-stroke change in identity one can undergo, providing further insight into not only the way in which identity is disrupted following stroke but how this disruption may continue and develop overtime. Moreover, the model suggests how a positive reconfiguration of post-stroke identity could be facilitated, if stroke survivors are supported sufficiently, it may be possible to encourage this positive reconfiguration and bring post-stroke liminality to an end. As such, this novel conceptualisation of stroke has many important implications for the delivery of stroke care.

This study suggests that to understand the post-stroke journey, and to encourage a positive reconfiguration of identity, the physical and psychosocial impact of stroke must not be treated as separate entities. Firstly, it is important to acknowledge that the way one physically interacts with the world is essential to the forming of identity. Gyllensten et al. (105) developed the theoretical concept of *embodied identity* to help explain how living in the body is directly tied to the way in which we live in relation to others and in society. It is through the body that one engages and experiences the world; thus, when this is limited, one's capacity to engage in the actions and roles that were important pillars of one's sense of self are reduced, restricting a person's ability to enact their identity in the physical world. This study supports the understanding that physical disruption is directly tied to psychological and social disruption, as the lived experience is inherently physical. This severance from participants' normal way of existing and being in the world was significant in triggering the psychological journey many survivors experienced post-stroke. On the other hand, this study adds further understanding to the way in which stroke can provoke a profound challenge to one's sense of self that often extends beyond the physical barriers placed by acquired impairments. While it is significant to understand the impact on stroke survivors' embodied identities, experiencing a stroke was seen as a major life-altering event for all participants, regardless of the severity of their physical impairments. Those who made successful physical and cognitive recoveries post-stroke reported experiencing an existential recalibration, as stroke had a profound impact on their personal and social identities. The variety of post-stroke experience captured in this study helps to showcase the truly biopsychosocial reality of long-term illness. We must not only be aware of the physiological, psychological, and social impact of illness, we must understand and address the way in which these three realms are interrelated and inherently intertwined in creating any individual's lived experience of illness if we are to successfully support them to provide person-centred care to positively rebuild their lives.

### Implications for the delivery of person-centred stroke care

The experiences of the participants have shown that their interactions with healthcare played a pivotal role in shaping, not only their initial experience, but their long-term adaption to living with stroke. If a patient-centred care approach is to be truly applied to the delivery of stroke care, the long-term adaption and recovery process can no longer be overlooked as key to the stroke survivor's journey and the seminal way in which identity transforms and adapts over time. This must be integrated and acknowledged from the very moment a stroke survivor contacts emergency services, to ensure that stroke care acknowledges the highly individual nature of a person's journey post-stroke. The conceptual model provides new insights into the factors that can facilitate or hinder recovery. HCPs must actively engage with patient narratives to flag these influencing factors in order to successfully provide, or signpost, tailored support.

Of central importance to experiences of post-stroke liminality and reconfiguring identity after stroke is the psychological journey a stroke survivor experiences following stroke. Without psychological support becoming a core, ubiquitous aspect of routine post-stroke support, the prevalence of survivors that struggle to escape a liminal sense of self will remain high and many may continue to struggle to frame stroke within their life narrative, helping them to give closure around the seismic impact stroke has had on their lives. Despite this, only 10.2% of patients in English, Welsh and Northern Irish hospitals with stroke received inpatient psychological support (106) (p. 29). Furthermore, a Stroke Association report found that 90% of stroke survivors in Northern Ireland believe their emotional and cognitive needs were not met once they left hospital (107). It is clear that a substantial gap remains in the provision of psychological support following stroke. This is important to address, as this study adds to the growing call for the significance of psychological support to be put front and centre when considering how best to encourage survivors to positively adapt to their life after stroke (60, 108–111). Moreover, as UK recommendations for stroke care, such as the Scottish Progressive Stroke Pathway, have highlighted the need for an increase in the quality and quantity of long-term psychological support after stroke (37, 38), ensuring that these are met is of critical importance. Significantly, these recommendations also acknowledge that all HCPs working with people after stroke must be aware of the centrality of the psychosocial impact of stroke and equipped to support survivors that are experiencing these challenges (37, 38). Greater awareness from all HCPs working with individuals following stroke is therefore required, as they must be receptive to the myriad of complex challenges to a survivor's sense of self, as the model indicates. In the case of this study, it can be seen how stroke survivors are often unaware and unprepared for the liminal status they may enter following discharge and the impact on their individual and social lives. If survivors are better prepared at pivotal stages of their recovery and rehabilitation, then it may be possible to limit the endurance of liminality and survivors may feel better equipped to reconfigure their identity positively following stroke. Moreover, there must be a shift in how rehabilitation is framed for survivors regarding their physical recoveries: it is less about physical functioning and more about physical being. How does one relate to the outside world, their friends and family, their occupation, with a limited body? The intersection of the physiological and psychosocial, and the centrality of identity between the two, must be acknowledged if we are to successfully support stroke survivors throughout their post-stroke journey.

Understanding, preparing, and supporting survivors for the multifaceted impact of stroke on their lives is of seminal importance. Stroke is not a chronic illness that exists within the confines of an individual, no matter how motivated and capable a survivor may be to recover, there will likely continue to be barriers that prevent their successful reintegration within society. As reflected in this study, when survivors attempt to rebuild their social identity, a major concern and difficulty is the possible inability to successfully return to employment post-stroke (25, 112–117). Previous studies have highlighted the important role of (re)gaining aspects of social identity are in regards to well-being and positivity post-stroke (21, 118). However, for many survivors following stroke, their impairments and limited capacity results in the erection of significant barriers to interacting socially and creating social value following stroke (119–122). While this has been evidenced within this study, what can also be seen is that re-integrating into society is a nuanced and complex process that is not purely defined by the physical and cognitive limitations an individual may possess post-stroke. It is therefore important that HCPs consider it to be their responsibility to support stroke survivors in their return to work, as recommended in the UK guidelines (37).

This study has highlighted the seminal role stigma plays on an individual, social, and societal level that creates an intricate landscape for stroke survivors to manage as they attempt to regain a valued position within society. Overall, there was often a notable difference in the social response to post-stroke impairments that was dependent on whether they were visible or invisible. For those with visible impairments, they could often be discounted through pity and being seen as a person who is not whole. For those with invisible impairments, they could be discredited by not being believed or having the extent of their impairments downplayed. This broadly aligns with Wainwright et al.'s (123) observations of misconceptions of stroke and the overgeneralisation that visible disabilities receives and the lack of acknowledgement of substantial challenges for invisible disabilities. What is also important to acknowledge is that as stroke survivors decide whether to disclose their stroke or not, many often grapple with an internalised sense of a devalued self (4, 6, 7, 11, 21, 75). This reality reflects Kulkarni's (124) exploration of workplace disclosure dilemmas for those with hidden disabilities and how the negotiation of disclosure can be as much an internal dilemma of self-image as an external struggle with the labels applied by others. The negative judgements and attitudes, both external and internal, stroke survivors could encounter often played a key role in restricting them from reintegrating into society, especially when attempting to return to employment. Without intervention, survivors are likely to be ill-equipped for the complex challenge stigma can pose to an individual as they rebuild their lives following stroke. The degrees of separation from society a survivor may experience is important to address as social isolation has been associated with worse cardiovascular and mental health outcomes (125). In contrast, being able to re-engage with society following stroke have been associated with positive physical and psychological outcomes, as well as improved well-being and quality of life (21, 126, 127). Therefore, the complex psychological and social nature of stigma must be addressed across the stroke pathway, as it is interwoven into the way in which survivors reflect upon and rebuild their identity.

It is important to note that, while this study was focused on the stroke survivors' narratives, experiences of liminality and a sense of lost or transitioning identity, will likely have an impact on a survivor's family and/or carers. Thus, ensuring that they are supported through this process is significant.

Finally, a core tenet of patient-centred care is the voice of the patient, however, it was clear that during interactions with HCPs that participants often felt there was a power imbalance, and that their voice held little weight. It is not possible to successfully deliver care which is intended to be centred around an individual, if the individual care is centred around is not listened to. Participants often spoke about how, when interacting with HCPs, they felt as if their voice was minimised or ignored. This reflects the findings of previous studies which have highlighted the limited agency stroke survivors can report having during their time receiving care (49, 128). What is important to acknowledge here is that, following stroke, many survivors have already experienced a significant change to their autonomy and, thus, their normalised sense of agency (129, 130). If their voice is minimised further within the boundaries of healthcare, this may reinforce the idea that their input and agency is worth less than it was prior to stroke. In the participants' experiences, a response to the power imbalance they have experienced can be seen, as they frequently identified taking control of their own health as a lesson many would advise future stroke survivors to heed. While expressing this position is empowering for survivors, it also indicates that some stroke survivors have lost trust and confidence in HCPs. This may not result in a total rejection of advice and support from HCPs; however, it adds to the disconnect many survivors can feel when they interact with HCPs (49). The potential outcomes of lost faith and changing perceptions in healthcare are indications that a truly person-centred approach to healthcare may still be lacking within some areas of stroke healthcare and that a more collaborative approach is required.

There are serious, direct implications for stroke survivors who feel their voice is not heard when interacting with HCPs, such as being misdiagnosed. Experiences of misdiagnosis are significant, as this directly puts stroke patients' lives and recoveries at risk (131). As participants noted in this study, misdiagnosis often occurred due to the expected symptoms of stroke, or due to a survivor's age. Here misdiagnosis becomes an even more pressing issue to address as stroke prevalence continues to increase and the average age of stroke continues to decrease (1, 132). Furthermore, as Venkat et al. (133) report, stroke misdiagnosis often occurred in patients who were commonly FAST-negative and displayed non-specific or atypical symptoms; this may extenuate pre-existing inequalities in the delivery of hyperacute care due to sex-specific differences of stroke being missed (134). For survivors of a certain age or sex, these barriers to receiving care can be compounded, as survivors risk of coming up against assumptions about the expected profile of stroke.

Participants also felt that their sense of self could often be at odds with the environments they were placed into. Many participants were already struggling with a challenge to their sense of identity following stroke, and being placed within a healthcare structure that is designed to cater to the elderly may help reinforce feelings of deficiency following stroke. These findings align with what previous studies have highlighted that being placed within stroke services that have a substantial elderly population can pose a threat to younger survivors' self-concepts and identities (135–138).

Difficulties interacting with healthcare also extended beyond the participants time within primary care. Indeed, it was noted that participants were often underprepared for the transition home and the decline in support they received. To help successfully manage these issues, there must be a consistent and person-focused continuity of care. However, it has been observed that survivors can encounter inconsistent and fragmented care throughout the stroke pathway, often defined by ineffective communication and information exchange (139, 140). Moreover, embedded around the conceptual model in Figure 1 are a myriad of individual and social factors that can both support and inhibit stroke survivors from successfully reconfiguring their identity, helping one to either exit a liminal space or remain within it. In doing so, further understanding of where extra support is needed to help support stroke survivors with their adjustment has been marked; this extends throughout a survivor's journey from hospitalisation to discharge to long-term adjustment, recovery, and social reintegration. This may provide avenues in which specific care can be targeted and to ensure that long-term support continues. Furthermore, to facilitate continuity in longer term support after stroke, the most recent National Clinical Guideline for Stroke for the UK and Ireland (37) recommends that stroke survivors be provided with a named healthcare professional (e.g., key worker) for further support and advice, as required.

In all, the possible lack of agency and dilution of voice stroke survivors may experience can been seen as a product of a concept Miranda Fricker (141) describes as *epistemic injustice.* This concept helps explain how knowledge claims can be unfairly dismissed, which leads to an actor's epistemic credibility and capacity being limited. In this study, epistemic injustice occurred when participants felt their input and voice was being unfairly discredited or overlooked by the HCPs they interacted with. To address the possible issues that arise from the culture that is embedded within healthcare, and the power imbalance this can create, one can turn to the concept of *epistemic humility*, which strives to recognise that one's expertise will always be bounded and that one must accept that, even with an expert status, they are also potentially fallible (142). Moreover, epistemic humility acknowledges that the creation of knowledge is a collaborative and interdependent activity (143). Integrating this approach within the healthcare setting will encourage HCPs to work collaboratively with patients. Participants reflected that, when such an approach was adopted, that it made them feel included within the decision-making process and that their voice was heard. To implement this effectively, healthcare must seek to go beyond the current understanding of patient-centredness, as stroke survivor's experiences, stories, and identities must be acknowledged as a vital and valid source of information; a different form of evidence that should shape the delivery of care and support that survivors receive. Dillon and Craig's (144) work seeks to highlight how gathering narrative evidence can complement and strengthen other, more scientific, forms of evidence. This study supports such an approach, as narrative evidence from service users should be taken into consideration to inform decision making, as part of a pluralistic evidence base underpinning clinical practice as well as clinical guidelines. Thus, epistemic humility and listening to stroke survivor stories must not stop at the level of individual HCPs interacting with survivors, there must be efforts to engrain these concepts within wider clinical guidelines, shaping stroke care into something which is truly person-centred.

### Limitations

This study was partially conducted during the COVID-19 pandemic. Naturally, the study had to be changed to remote-only. Ten face-to-face interviews were conducted prior to the pandemic, which had implications for the recruitment of participants. The 10 participants interviewed prior to the pandemic had a mean age of 63 years old; the 20 participants interviewed after the start of the pandemic had a mean age of 50. This was likely due to the adapted recruitment strategy targeting online avenues, and the online and social media advertisements having the greatest success at returning potentially interested participants. Initially, the study aimed to speak to residents in care homes. Two were spoken to prior to the COVID-19 pandemic, however, following the outbreak, and the severity at which care homes were affected by the pandemic in the UK, meant recruiting further participants was not possible. Furthermore, while the recruitment strategy was expanded to cover the whole of the UK post-pandemic, participants were only recruited from Scotland (13) and England (17), as no responses were recorded from Northern Ireland or Wales.

Furthermore, there was a bias towards participants from more urban environments than rural. This is important as those who live in more rural environments may have difficulty accessing important healthcare and support services (144–146). Moreover, while stroke survivors with aphasia were interviewed, none indicated they required the communicative device, suggesting they did not have severe communicative impairment. This is an important demographic to contact as severe aphasia can have a substantial impact on a stroke survivors emotional well-being and ability to participate socially following stroke (147, 148). Furthermore, while a range of ages was captured in this study, there was difficulty recruiting male stroke survivors aged under 40 or any stroke survivor aged 18–30. This study also does not address the varied experiences people of different racial or ethnic backgrounds will likely experience, and no marginalised voices for those who may be migrants or homeless were captured. Finally, no stroke survivors who were knowingly approaching their end stage of life, these participant experiences would likely be rather different and sit in contrast to those captured within this study.

### Implications for future education, practice, and research

As highlighted within this study, and within the latest UK and Ireland stroke guidelines and recommendations (37, 38), there is need for further HCP training and education that highlights the varied psychosocial factors that influence identity reconfiguration after stroke and the need to adapt patient-centred care so that long-term identity change is at the heart of the stroke care that is delivered. Through heightening HCP awareness and understanding of these issues, HCPs may be able to better support survivors as they begin and continue to adjust to the long-term impact of stroke on their lives. What is needed now, is the design and implementation of educational resources that help to achieve this change in attitudes, awareness, and empathy in HCPs and within the delivery of patient-centred care. The research team has continued this line of work, through a study that sought to co-create digital stories with stroke survivors with the aim of synthesising collective lessons from individual experiences of interacting with healthcare professionals, further strengthening the call for such educational resources for HCPs working with people after stroke (149). The co-created lessons and digital stories can be found at: https://lifeafterstroke.webflow.io/.

Overall, the centrality of an individual's long-term psychosocial adjustment post-stroke has been identified and this study adds to the growing call for this issue to be a central focus of support and research in future (37, 38, 60). The concept of liminality, the conceptual model, and other key findings of this research can be built upon, where the focus of future research should be centred around widening and deepening our understanding of experiences of liminality and the impact on a survivor's post-stroke agency in order to better understand how we can support a positive reconfiguration of identity and reintegration into society.

## Conclusion

The model developed through this constructivist Grounded Theory study interviewing thirty UK stroke survivors provides a novel conceptualisation of the challenge to identity post-stroke and a comprehensive theoretical map that explains what factors help or hinder stroke survivors to reconfigure their identity positively following stroke. The novel application of liminality provides new insights to explain the transitional nature of post-stroke identity, and the uncertainty many can feel as they are suddenly caught between pre- and post-stroke identities. This could result in an evolving and enduring period of liminality, a state of existence defined by uncertain identity. The furthered understanding of the challenge for stroke survivors to rebuilding identity post-stroke has evidenced how this process is evolving, long-term, and potentially indefinite. The study's findings, and the resultant conceptual model, have important implications for the delivery of person-centred stroke care. This work reinforces the call for psychological well-being and support to be made a priority within stroke care. Moreover, it calls for the stroke survivor, their identity, and the long-term challenges to their sense of self to be the focal point of the delivery of person-centred stroke care that is delivered consistently from initial stroke onset right through to long-term adjustment post-stroke. The physical, psychological, and social realities of each survivor must be taken in to account to truly understand and address the long-term impact of stroke on identity.

## Data Availability

Existing datasets are available in a publicly accessible repository: Publicly available datasets were analyzed in this study. This data can be found here: https://researchonline.gcu.ac.uk/files/91788126/91788075.pdf. Further inquiries can be directed to the corresponding author.
