# Supplementary material for: Stroke and liminality: narratives of reconfiguring identity after stroke and their implications for person-centred stroke care
Source: Front Rehabil Sci. 2024 Dec 3;5:1477414. doi: 10.3389/fresc.2024.1477414 (PMC11651291; doi:10.3389/fresc.2024.1477414)
Supplement: Supplementary file 1 [file Datasheet1.docx]

**Stroke and Liminality: narratives of reconfiguring identity after stroke and their implications for person-centred stroke care - Interview Question Guide**

*Authors*: Joseph Hall, Frederike van Wijck, Thilo Kroll, Helena Bassil-Morozow

Before stroke

- Take me to the time before the stroke.
- What was a normal day like for you before the stroke?
- What was most important to you before the stroke?

Hospital (potentially ask about late in interview due to sensitivity)

- Would you mind telling me about your time in hospital?
- How did your family (friends) respond to your initial hospitalisation?
- What gave you hope while you were in hospital? What was frustrating?

Returning Home

- Can you tell me what it was like to return home after the stroke?
- How did you find the adjusting to life at home?

Life Now

- What is a normal day like for you now?
- How has your life changed?
- What were you surprised about during your recovery? (What helped you? What didn’t help you?)
- What impact has stroke had on your life?
- Are there any positive changes you have noticed or experienced following the stroke?
- In what ways have your relationships been affected following stroke?
- What do you think you have learned?

**Further In-depth Questions**

**I Before Stroke:**

What was a normal day like for you before the stroke?

- What is the normal day for you now?

What was important to you before your stroke?

Did you work before the stroke?

- If so, what did you do?
- Do you still work?

**II Hospitalisation (don’t foreground):**

What were you thinking about most while at hospital?

How did your family cope with your hospitalisation?

Before leaving hospital, did you feel you had recovered well?

**III Returning Home**

What challenges did you face when returning home?

- How did you deal with the challenges you faced?

How did others help you with these challenges?

What do you feel had changed the most for you?

What was most important to you during your rehabilitation?

- Did you feel you were listened to?

**IV After Stroke:**

What were you most worried about following the stroke?

- Has this changed over time?

Is there anything you find frustrating following the stroke?

- How do you manage frustrating situations that are a result of the stroke?

How do you think your recovery has gone?

- Is there anything you are surprised about?

How do you feel you have adjusted to life following the stroke?

- If not well, what do you think are the barriers stopping you?
- If well, what do you think helped you most to do this?

What do you think is most important in helping someone recover from stroke?

- If you were to give advice to someone who had just had a stroke, what would it be? (carer, friends, family and healthcare professionals)

What advice would you give someone that could help them support a family member who has had a stroke?

How did your family cope after the stroke?

In what way were your friendships affected after the stroke?

- Has the way you socialise with your friends changed?

Do you feel comfortable leaving the house?

- If not, what are the biggest issues you face when leaving the house?
- How do others see your impairments?

Have you had to change the way you interact with others following stroke?

How do you think others see you since your stroke, is it different compared to before your stroke?

- Tease out issues

Do you feel connected to your local community?

- If so/not, how?

What matters to you now? Has it changed from what mattered to you in the past?

- What do you value most in life? Has it changed?
- What are you grateful for?

Have you learned anything about yourself that you did not know?

- Do you believe stroke has changed how you view yourself as a person?

What brings you most joy?

- How has this changed?

What have you learned following your experience?

- Have you learned anything new?
- Have your aspirations changed because of the stroke?

Have your priorities changed following stroke?
